# Anti-proliferative Effect of C3 Exoenzyme in Fibroblasts is Mediated by c-Jun Phosphorylation

**DOI:** 10.5334/1750-2187-12-1

**Published:** 2017-04-03

**Authors:** Leonie von Elsner, Sandra Hagemann, Ingo Just, Astrid Rohrbeck

**Affiliations:** 1 Institute of Toxicology, Hannover Medical School, Hannover, DE

**Keywords:** C3 exoenzyme, MAPK signaling, p38, c-Jun, cell proliferation

## Abstract

The ADP-ribosyltransferase C3 exoenzyme from *C. botulinum* selectively inactivates Rho and is therefore often used as an inhibitor for investigations on Rho signaling. Previous studies of our group revealed that C3 inhibited cell proliferation in HT22 cells accompanied by increased transcriptional activities of Sp1 and c-Jun and reduced levels of cyclin D1, p21 and phosphorylated p38. By use of a p38α-deficient and a p38α-expressing control cell line, the impact of p38 on C3-mediated inhibition of cell proliferation and alterations on MAPK signaling was studied by growth kinetic experiments and Western blot analyses. The cell growth of p38α-expressing cells was impaired by C3, while the p38α-deficient cells did not exhibit any C3-induced effect. The activity of the MKK3/6-p38 MAPK signaling cascade as well as the phosphorylation of c-Jun and JNK was reduced by C3 exclusively in the presence of p38α. Moreover, the activity of upstream MAPKKK TAK1 was lowered in the p38α-expressing cells. These results indicated a resistance of p38α-deficient cells to C3-mediated inhibition of cell growth. This anti-proliferative effect was highly associated with the decreased activity of c-Jun and upstream p38 and JNK MAPK signaling as a consequence of the absence of p38α in these cells.

## Introduction

The bacterial C3 exoenzyme (C3) from *Clostridium botulinum* selectively inactivates RhoA, RhoB and RhoC by ADP-ribosylation [1,2,3], resulting in various cellular consequences, such as morphological alterations due to the disturbance of actin cytoskeleton and formation of contractile ring [4,5,6]. Therefore, C3 is often used as cell-biological tool for deciphering the function of Rho in cell signaling cascades. Additionally, C3 increases enzyme-independently the axonal growth and branching in primary hippocampal neurons [7]. Besides this neurotrophic effect, C3 effects apoptosis induction in various cell types. On the one hand, C3 promotes apoptosis in lymphocytes and HUVEC cells, but on the other hand prevents HT22 and retinal ganglion cells from cell death after apoptosis induction *in vitro* and *in vivo* [8,9,10,11,12].

Previous studies also identified an anti-proliferative effect of C3 in various cell types [12,13,14]. Both, C3 and the enzyme-deficient mutant C3-E174Q, reduced the level of cell cycle regulator cyclin D1, increased the abundance of the negative cell cycle regulator RhoB and inhibited cell proliferation with varying extent in the murine hippocampal HT22 cells [12,15]. Furthermore, our group demonstrated a strong correlation between C3-induced inhibition of cell growth and the increased transcriptional activity of Sp1 accompanied by an elevated level of p21 in HT22 cells [16]. Besides cyclin D1 and p21, mitogen-activated protein kinases (MAPK), such as ERK and p38 as well as c-Jun, the substrate of the c-Jun N-terminal kinase (JNK), play a crucial role in the regulation of cell proliferation [17,18,19,20,21]. The main activation of MAPK canonically occurs via phosphorylation by upstream MAPK kinases, which in turn are phosphorylated and activated by MAPKK kinases such as MEKKs, ASK1 and TAK1 [22,23,24,25,26,27,28].

Previous studies revealed that C3 reduces the level of phosphorylated p38 and increases the phosphorylation of c-Jun in HT22 cells as well as activated ERK in C3-treated primary hippocampus neuron cultures after 48 h [16,29]. Additionally, a combined incubation of HT22 cells with p38 inhibitor skepinone-L and C3 causes a slightly additional anti-proliferative effect compared to the solely treatment with C3 indicating an involvement of p38 in the C3-induced growth inhibition [16].

For a detailed determination of the influence of p38 on the C3-mediated anti-proliferative effect, two cell lines, a p38-deficient mouse embryonic fibroblast (MEF) and a p38-reconstituted MEF cell line, were applied in this study. These cells were prepared as p38 ^–/–^ MEFs from embryos obtained from the crossing of heterozygote p38α ^+/–^ mice [30]. p38α represent the mainly expressed p38 isoform whose knockout results in lethality of p38α-deficient embryos [31,32]. For convenience the p38α is abbreviated as p38 in the following. For comparison of this p38-deficient with a p38-reconstituted cell line, the p38 ^–/–^ MEF cells were transfected either with an empty vector (EV) or with a vector encoding for p38α and were immortalized by use of simian virus 40 large T-antigen [33]. Therefore, the applied p38-deficient p38 ^–/–^ EV MEF cells were solely deficient of p38α, but still contain the genetic information of the other p38 isoforms p38β, p38γ and p38δ. In contrast, the control cells p38 ^–/–^ p38 MEFs possess the genetic information of all isoforms.

In this present study, we aimed to define the influence of p38 on the inhibition of cell proliferation induced by C3 by use of a p38-deficient cell line. Interestingly, growth kinetic experiments demonstrated that C3 impaired only the cell proliferation in the presence of p38 (p38 ^–/–^ p38 MEFs), but not in p38-deficient cells. Moreover, C3 affected not only the MKK3/6-p38 signaling, but also the JNK-c-Jun pathway and upstream MAPKKK TAK1 exclusively in the presence of p38. Thus, these results elucidated that C3 altered distinctly the MAPK signaling by inducing the inhibition of cell proliferation in the p38 ^–/–^ p38 MEFs.

## Materials and methods

### Cell culture

The p38α-deficient p38 ^–/–^ EV and p38α-reconstituted p38 ^–/–^ p38 MEFs were a kind gift from Prof. Matthias Gaestel (Hannover Medical School, Germany). The cells were cultivated in Dulbecco’s modified essential medium (Sigma-Aldrich Chemie GmbH, Munich, Germany) with 5% fetal bovine serum (PAN Biotech GmbH, Aidenbach, Germany), 1% penicillin, 1% streptomycin (PAA Laboratories GmbH, Pasching, Austria), 1 mM sodium pyruvate (Biochrom AG, Berlin, Germany) and 1 mM MEM None-essential amino acids (Gibco, Life Technologies, Paisley, UK) at 37°C and 5% CO_2_. When the cells reached confluence, they were passaged.

### Growth kinetics

For growth kinetics experiments 30,000 cells mL^–1^ were seeded onto 3.5 cm-plates (Sarstedt AG & Co, Nürnbrecht, Germany). The next day, the cells were treated with medium for control conditions, 500 nM C3 or C3-E174Q. Every 48 h the medium was replaced including C3 or C3-E174Q. At respective time points the cell number was determined as described previously [12].

### Western blot analysis

For western blot analyses of the incubation times less than 72 h 150,000 cells mL^–1^ were seeded onto 3.5 cm-plates. For later time points 30,000 cells mL^–1^ were utilized. The next day, cells were treated with medium as control, 500 nM C3 or C3-E174Q for various incubation times. At defined time points the cells were washed with ice-cold PBS and frozen at –20°C. Preparation of cell lysates and Western blot analyses was performed as described previously [12]. The samples were applied on several 15% sodium dodecyl sulfate (SDS)-gels simultaneously for studying different proteins with the similar molecular weight in the same experimental attempt. Proteins with a higher molecular weight, such as TAK1 and phospho-TAK1, were examined by SDS-PAGE of 10% SDS-gels. For analysis of phosphorylated proteins 1 mM sodium-ortho-vanadate (Sigma-Aldrich Chemie GmbH, Munich, Germany) was applied in lysis buffer and the preparations were carried out on ice. The following primary antibodies were applied for immunoblotting: α-RhoA, α-p38, α-JNK1 (Santa Cruz Biotechnology, CA, USA), α-cyclin D1, α-ERK1/2, α-p-AKT Ser473, α-p-p38 Thr180/182, α-p-c-Jun Ser63, α-p-MKK3/MKK6 Ser189/207, α-p-SAPK/JNK Thr183/Tyr185 (Cell Signaling Technology, Danvers, MA, USA), α-p-MKK4 Ser257/Thr261, α-TAK1 (R&D Systems Inc., Minneapolis, MN, USA), α-AKT/PKB, α-p-TAK1 Ser412 (Merck Millipore KGaA, Darmstadt, Germany), α-pp-ERK1/2 Thr183/Tyr185 (Sigma-Aldrich Chemie GmbH, Munich, Germany), α-GAPDH (Zytomed Systems GmbH, Berlin, Germany) and ADP-ribosylated RhoA/B was determined by the monoclonal ViF140_A1-hFc-antibody kindly provided by Viola Fühner and Michael Hust (Technische Universität Braunschweig, Germany; described in [34]). The chemoluminescence reaction was performed by ECL Femto (Pierce, Thermo Fisher Scientific Inc., Rockford, IL, USA) and the signals were detected by Chemostar software (Intas Science Imaging Instruments GmbH, Göttingen, Germany) for the remaining blots. The densitometric analysis was executed by Kodak 1D software (KODAK GmbH, Stuttgart, Germany).

### Immunofluorescence

The cells were seeded onto HCl-treated coverslips. After 24 h the cells were treated with 500 nM C3 at 37°C and 5% CO_2_. After the indicated incubation times the following staining was performed at room temperature. The cells were stained as described previously [35].

### Expression and purification of recombinant C3 proteins

C3 wildtype and C3-E174Q proteins were produced as recombinant GST-fusion proteins. Therefore the plasmid pGEX-2T (gene of *Clostridium botulinum* C3, accession no. X59039) was transferred into *E.coli* TG1 followed by protein expression and purification as described previously [12].

### Reproducibility of the experiments and statistics

All experiments were performed independently as biological replicates at least three times. The graphical and statistical analysis was performed by use of Microsoft® Excel 2010 version 14.0 (Microsoft Corporation, Redmond, USA) and GraphPad Prism version 6.07 (GraphPad Software, Inc., San Diego, CA, USA, 2015). The figures depict results from representative experiments with mean values (n ≥ 3) ± SEM. For calculation of statistical significance of differences between treated compared to untreated cells a two-sided unpaired Student‘s *t* test and one-way ANOVA were applied (* = p ≤ 0.05, ** = p ≤ 0.01 and *** = p ≤ 0.001).

## Results

### Comparable uptake of C3 in both cell lines

To check comparability of C3 uptake into both MEF cell lines, Western blot analyses of ADP-ribosylated Rho were applied. As shown in Figure S1, ADP-ribosylated RhoA was detected in both C3-treated cell lines already after 2 h. Notably, ADP-ribosylation altered the migration behavior of the modified RhoA in SDS-PAGE causing a shifted RhoA band detectable by RhoA antibody on the blot (upper panel). On the contrary, the antibody against ADP-ribosylated RhoA/B (VIF140_A1-hFc) detected only the ADP-ribosylated form of RhoA/B, but not unmodified RhoA/B (middle panel). The enzyme-deficient mutant C3-E174Q served in all experiments as control to validate the Rho-dependence of the observed findings. In contrast, the C3-mediated characteristic alterations of cell morphology in terms of the actin cytoskeleton reorganization and the appearance of multi-nuclear cells were not significantly increased after 2 h (Figure S2). Nevertheless, in both cell lines, the manifestation of such morphological changes were comparable and prolonged until 120 h. Overall, a comparable shift of RhoA band and band intensity of ADP-ribosylated Rho after 2 h imparted an identical p38-independent uptake of C3 in both cell lines, allowing further studies concerning the C3-mediated impact on proliferation and various signaling cascades.

### C3 impaired cell growth independently of cyclin D1, ERK and AKT

**Figure 1:**
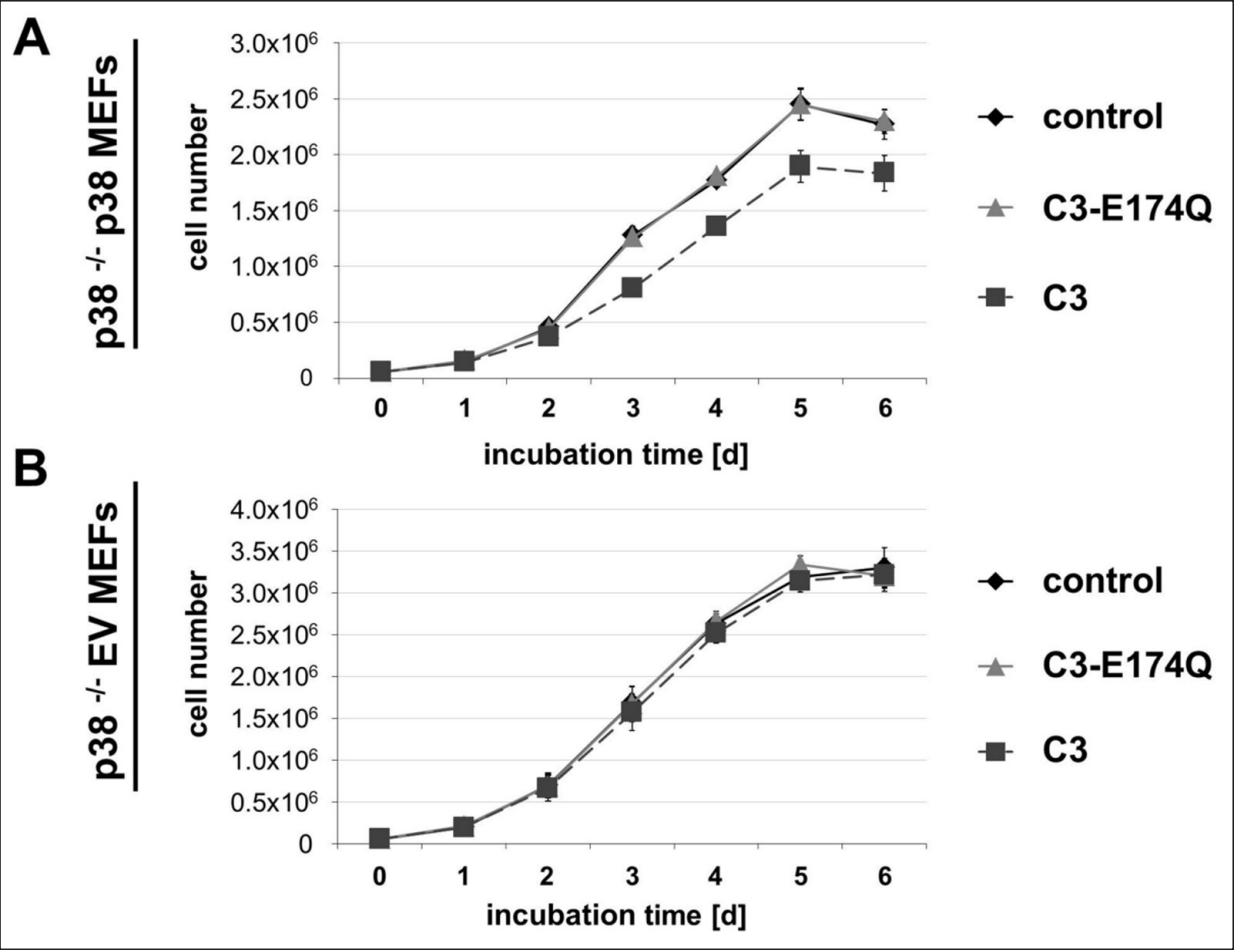
Influence of C3 on cell proliferation. For determination of the influence of C3 on cell proliferation, growth kinetic experiments were performed with both cell lines. p38 ^–/–^ p38 MEFs **(A)** or p38 ^–/–^ EV MEFs **(B)** were treated with 500 nM C3 or 500 nM C3-E174Q by replacing the medium including C3 or C3-E174Q every 48 h. The cell number was determined by trypan blue counting assay in duplicate after indicated incubation times. Growth curves represent mean values ± SEM of independent experiments (n = 3).

To examine the influence of p38 on the C3-mediated anti-proliferative effect, growth kinetic experiments of both cell lines were performed (Figure 1). C3 impaired the proliferation of p38 ^–/–^ p38 MEFs starting from the second day of treatment, whereas C3-E174Q did not exhibit any effect (Figure 1A). In contrast, neither C3 nor C3-E174Q affected the cell proliferation of p38-deficient p38 ^–/–^ EV MEFs (Figure 1B). The flattening of the growth curves of both cell lines after 5 d implied a contact inhibition-mediated impact on cell proliferation at this time point.

In hippocampal HT22 cells, C3 and C3-E174Q reduced the abundance of cell cycle regulator cyclin D1 resulting in an inhibition of cell proliferation [12]. Therefore, we studied the influence of C3 on cyclin D1 (Figure S3). In contrast to the results of the C3-treated HT22 cells, no significant alteration of the abundance of cyclin D1 was detectable in both cell lines after the indicated C3 or C3-E174Q incubation. Moreover, the MAP kinase ERK and AKT are known to be involved in the regulation of cell proliferation [17,36]. No significant alterations of the phosphorylation of ERK and AKT were observable in both cell lines after the incubation with C3 or C3-E174Q (Figure S4).

### Activation of MKK3/6-p38-signaling pathway

**Figure 2:**
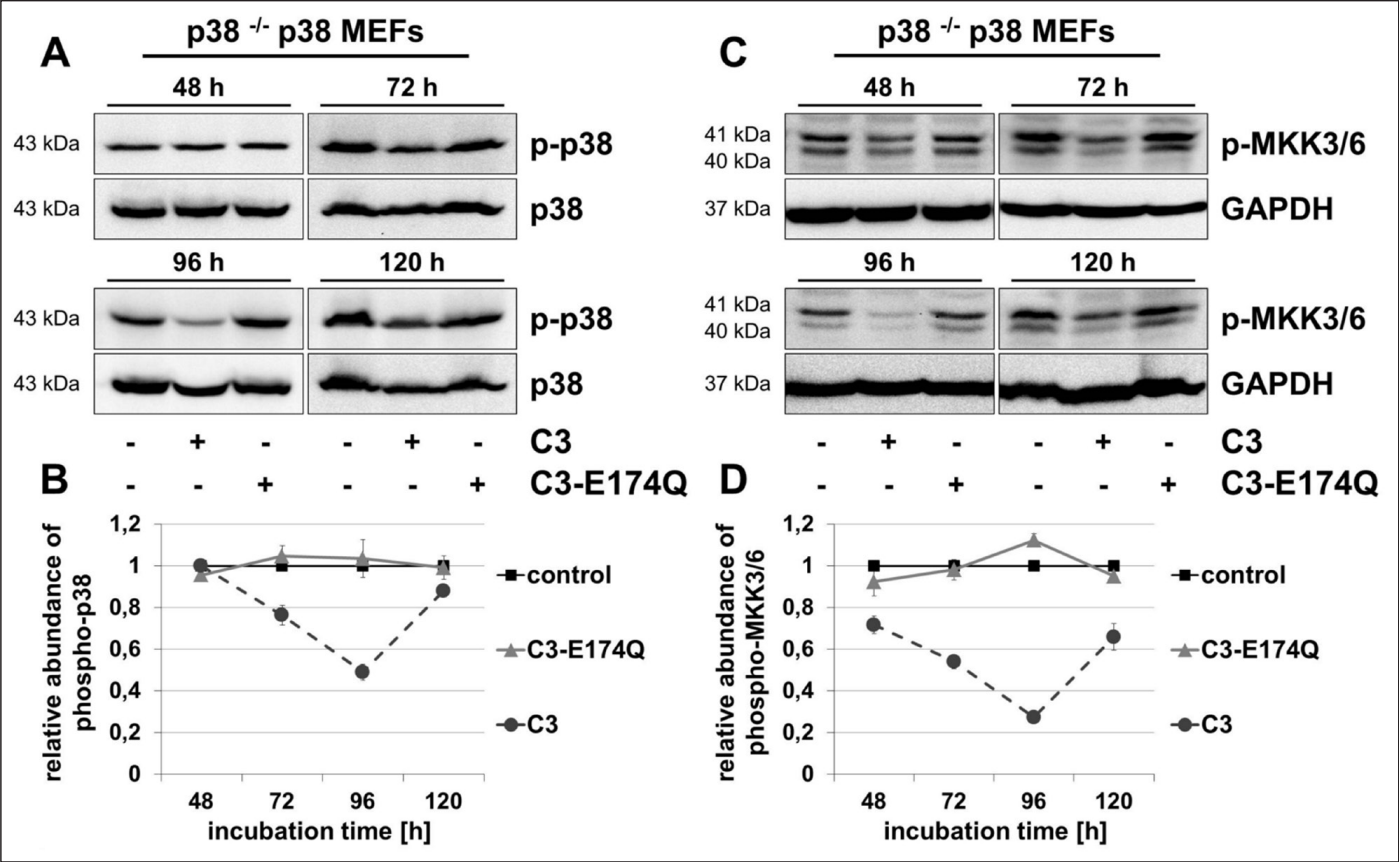
Reduced phosphorylation of p38 and MKK3/6 in C3-treated p38 ^–/–^ p38 MEFs. p38 ^–/–^ p38 MEFs were incubated with 500 nM C3 or 500 nM C3-E174Q for indicated time points, lysed and submitted to Western blot analyses for phospho-p38 (p-p38) and p38 **(A, B)**, or phospho-MKK3/6 (p-MKK3/6) and GAPDH **(C, D)**. **(B)** For densitometric quantification of p-p38, the signal intensity of p-p38 was adjusted to the corresponding intensity of p38. **(D)** The densitometric analysis of p-MKK3/6 was performed by normalizing the signal intensity of p-MKK3/6 of C3- and C3-E174Q-treated cells to the signal intensity of control cells. Representative Western blot analyses are illustrated. Results represent mean values ± SEM of independent experiments (n = 3).

Next, the influence of C3 on MKK3/6-p38 signaling was determined by Western blot analyses. In p38 ^–/–^ p38 MEFs, C3 decreased significantly the phosphorylation of p38 from 20% after 72 h to 50% after 96 h (Figure 2A, B). After C3-treatment for 120 h the level of phospho-p38 reconstituted to a marginal reduction of about 10%.

Upstream of p38, the MAPK kinases MKK3/6 activate p38 by phosphorylation at the amino acid residues Thr180 and Tyr182 [22,26]. Incubation of p38 ^–/–^ p38 MEFs with C3 significantly decreased the level of phospho-MKK3/6 time-dependently from 30% after 48 h to 70% after 96 h (Figure 2C, D). After 120 h the phosphorylation of MKK3/6 maintained at a reduced level of 30%. Only a marginal reduction of phospho-MKK3/6 was detectable after incubation with C3 for 48 and 96 h in p38 ^–/–^ EV MEFs (Figure S5B, C). C3-E174Q did not affect phosphorylation of p38 and MKK3/6 in both cell lines at any times.

### Activation of MKK4-JNK-c-Jun-signaling pathway

**Figure 3:**
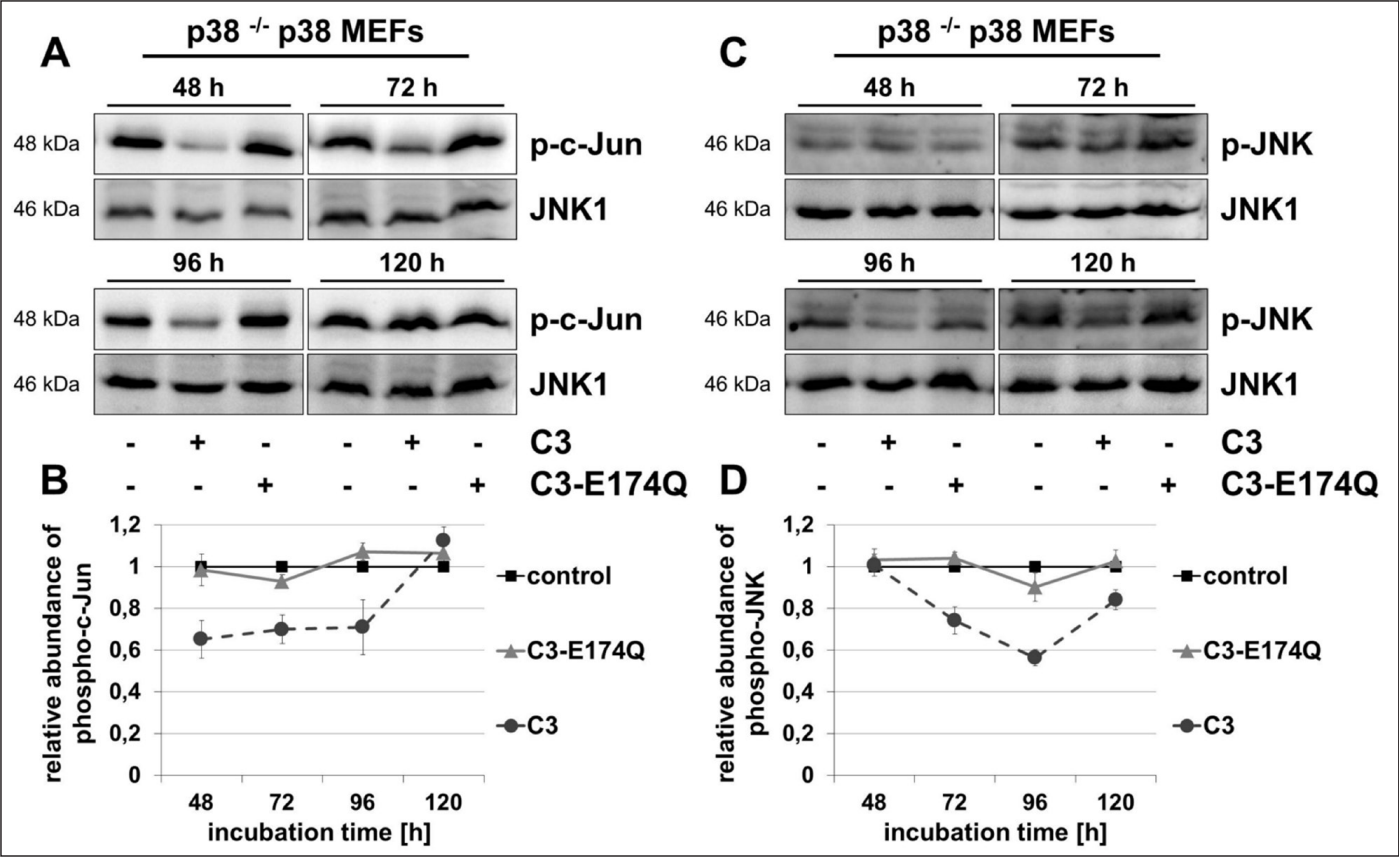
C3-induced decreased phosphorylation of c-Jun and JNK in p38 ^–/–^ p38 MEFs. p38 ^–/–^ p38 MEFs were incubated with 500 nM C3 or 500 nM C3-E174Q for indicated time points. Cells were lysed and submitted to Western blot analyses for phospho-c-Jun (p-c-Jun) **(A, B)**, or phospho-c-Jun N-terminal kinase (p-JNK) and JNK1 **(C, D)**. For densitometric analysis the signal intensity of p-c-Jun **(B)** or respectively p-JNK **(D)** was normalized to the corresponding intensity of JNK1. Representative blots are illustrated. Results depict mean values ± SEM of independent experiments of p-c-Jun (n = 3) and p-JNK (n = 4).

Due to the strong involvement of c-Jun in the regulation of cell proliferation, also the activity of the MKK4-JNK-c-Jun pathway was studied [20,21]. The phosphorylation of c-Jun was decreased by 30–35% between 48 and 96 h in C3-treated p38 ^–/–^ p38 MEFs (Figure 3A, B). After 120 h the level of phospho-c-Jun was comparable to control.

The transcriptional activity of c-Jun is activated by JNK by phosphorylation at Ser63 and Ser73 [37,38,39]. For determining the activation of JNK by phosphorylation, the main focus was on the predominant bands at 46 kDa depicting the major splicing variants of JNK1 that is also primary responsible for transcriptional activation of c-Jun by phosphorylation [39,40,41]. Because of the lack of significant effects (data not shown), the JNK isoforms at 54 kDa (p54) were neglected, just as an additional unspecific band at about 35 kDa. Incubation of p38 ^–/–^ p38 MEFs with C3 significantly decreased phosphorylation of JNK from 72 to 120 h with a maximal reduction of the phospho-JNK abundance by 40% after 96 h (Figure 3C, D). In contrast, C3-E174Q did not affect the level of phospho-JNK. No effects on phospho-c-Jun or phospho-JNK were detected in p38 ^–/–^ EV MEFs (Figure S6). Overall, in these cells the level of phospho-JNK appeared to be lower compared to the p38 ^–/–^ p38 MEFs.

**Figure 4:**
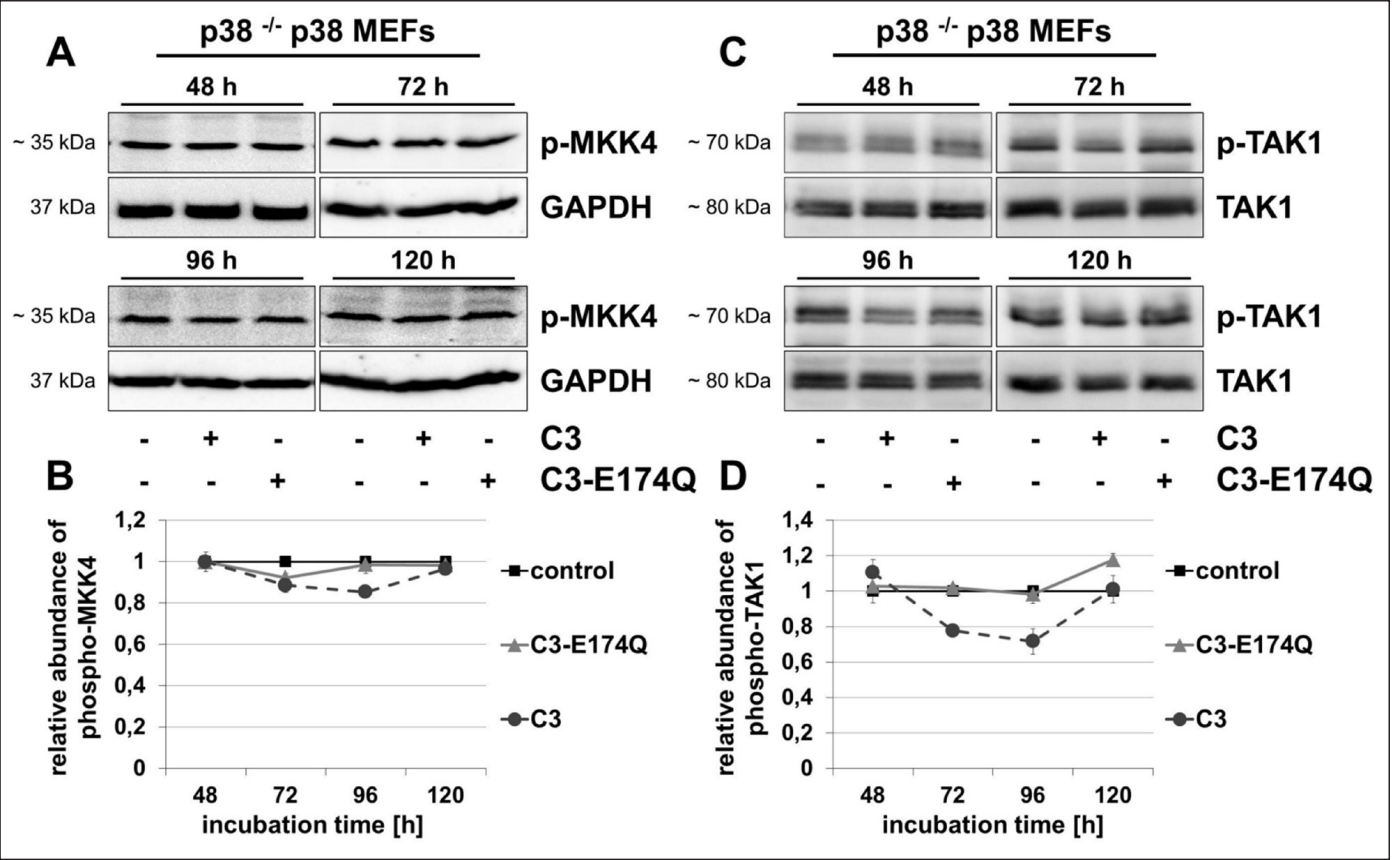
C3 marginally affected phospho-MKK4 and reduced the level of phospho-TAK1. After treatment of p38 ^–/–^ p38 MEFs with 500 nM C3 or 500 nM C3-E174Q for indicated incubation times, cells were lysed and applied to Western blot analyses for phospho-MKK4 (p-MKK4) and GAPDH **(A, B)**, or phospho-TAK1 (p-TAK1) and TAK1 **(C, D)**. **(B)** For densitometric quantification of p-MKK4 the signal intensity of p-MKK4 of C3- or C3-E174Q-treated cells were adjusted to the corresponding intensity of untreated control cells. **(D)** For quantification of p-TAK1 the signal intensity of p-TAK1 were normalized to the signal intensity of TAK1. Representing Western blot analyses are shown. Results illustrate mean values ± SEM of independent experiments (n = 3).

The MAPK kinases MKK4 and MKK7 are known to activate JNK by phosphorylation at residues Thr183 and Tyr185 [22,24,42,43]. Western blot analyses revealed only marginal, negligible effects of C3 and C3-E174Q on the phosphorylation of MKK4 in both cell lines (Figure 4A, B and Figure S6C, D).

### Influence of C3 on MAPKK kinase TAK1

Therefore, the influence of C3 on a MAPKK kinase that was able to regulate both, the p38- and JNK-pathway, was examined. TAK1 is upstream of various MAPKKs and activates MKKs, such as MKK3, MKK4, MKK6 and MKK7 [25,26,44,45,46]. As many other MAPKK kinases, TAK1 is activated by phosphorylation at different phosphorylation sites. Incubation of p38 ^–/–^ p38 MEFs with C3 significantly reduced the phosphorylation of TAK1 by 20–30% after 72 and 96 h (Figure 4C, D), whereas the marginal effects of C3 and C3-E174Q in p38 ^–/–^ EV MEFs were negligible (Figure S7A, B).

## Discussion

Previous studies on hippocampal HT22 cells revealed that C3 as well as C3-E174Q strongly inhibit cell proliferation starting at day two accompanied by a decreased abundance of cyclin D1 [12]. Contrary to that, C3 did not influence the proliferation in p38-deficient p38 ^–/–^ EV MEFs and only caused a moderate inhibition of cell proliferation in p38 ^–/–^ p38 MEFs. Moreover, the abundance of cyclin D1 was not altered by C3 in both MEF cell lines. Thus, these results imply that the C3-mediated inhibition of cell proliferation in p38 ^–/–^ p38 MEFs strictly depended on RhoA inactivation and the presence of p38, but was independent of cyclin D1. Furthermore, neither ERK nor AKT signaling were involved in this anti-proliferative effect. The enzyme-deficient C3-E174Q did not cause any effect in both cell lines concerning the cell proliferation or respective protein abundances of studied signaling pathways. This emphasizes that the C3-induced anti-proliferative effect and alterations on signaling pathways were strictly Rho-dependent in p38 ^–/–^ p38 MEFs in contrast to HT22 cells.

Hence, further MAP kinase signaling pathways were analyzed for identifying possible cascades involved in the C3-catalyzed impairment of cell proliferation in p38 ^–/–^ p38 MEFs. As demonstrated by the lack of C3-mediated anti-proliferative effect in p38-deficient cells, the p38 signaling pathway was principally involved in the regulation of cell proliferation with Rho as upstream regulator.

**Figure 5:**
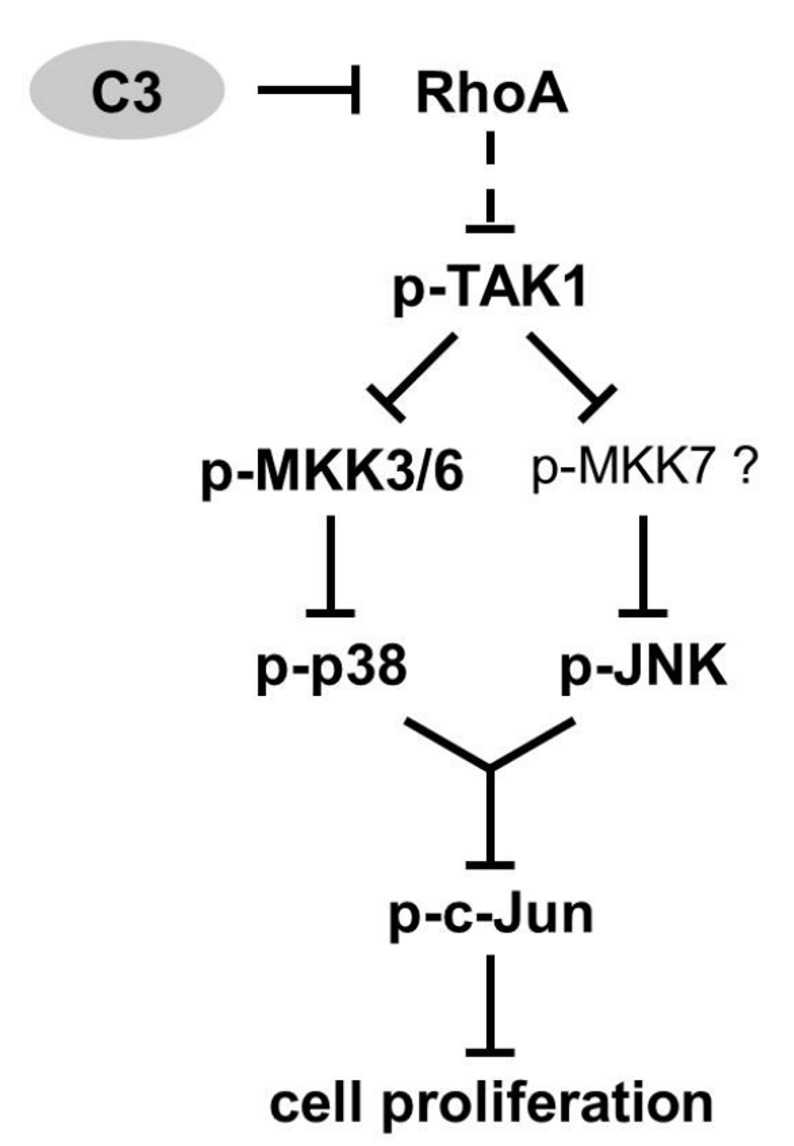
Proposed mechanism of C3-induced growth impairment. As a consequence of Rho inactivation by C3, the activity of MAPKKK TAK1 and downstream MKK3/6-p38 or respectively JNK pathway was decreased p38-dependently in p38 ^–/–^ p38 MEFs. Thus, the phosphorylation of c-Jun was reduced resulting in an impaired cell proliferation in C3-treated p38 ^–/–^ p38 MEFs.

Similar to the results in gastric pariental cells and HT22 cells, C3 reduced phosphorylation of p38 in p38 ^–/–^ p38 MEFs after 72 and 96 h [16,47]. Several studies suggested that an interaction of phospho-TAK1 and TAB1 positively regulate the basal cytoplasmic level of phospho-p38α by a canonical MAPK pathway signaling via a TAK1-mediated activation of MKK3/6 and an enhanced MKK-independent, TAB1-induced autophosphorylation of p38α [26, 48,49,50,51,52]. Concerning the results of p38 ^–/–^ p38 MEFs, this model agrees with the observed reduced phosphorylation of TAK1, MKK3/6 and p38 by confirming a canonical signaling pathway (Figure 5). Certainly, the delayed, reduced phosphorylation of TAK1 after 72 h compared to the decreased phosphorylation of MKK3/6 after 48 h indicates the involvement of another C3-affected, p38-dependent MAPKKK upstream of MKK3/6. In this context, both, JNK and p38 cascades are also activated by alternative MAPKKKs, such as ASK-1, MLK-2/3 and MEKK1-4 [27,28,53,54,55,56,57,58]. Notably, the impact of C3 on other MAPKKK and phosphatases that are able to influence the MKK3/6-p38 signaling was not investigated and remained unknown.

The associated appearance of the C3-mediated reduced abundance of phospho-c-Jun and the impairment of cell proliferation exclusively in p38 ^–/–^ p38 MEFs assumes a direct correlation between these two effects. Similar to p38, the role of c-Jun in cell proliferation is controversal and cell type-specific [16,18,19,20,21,59,60,61]. Various kinases, such as JNK, ERK, p38, GSK3 or cyclin-dependent kinase-3 are able to regulate the phosphorylation of c-Jun, but the lack of C3-induced effects on ERK and AKT ruled out their involvement [37,39,62,63,64]. In this current study, p38 was required for C3-mediated decrease of phospho-c-Jun, since C3 had no effect in p38-deficient MEFs. In accordance with that, activated p38 phosphorylates c-Jun *in vitro* [64]. Certainly, the reduction of c-Jun phosphorylation arose after 48 h, whereas the decreased level of activated phospho-p38 was firstly detected after 72 h in p38 ^–/–^ p38 MEFs. This indicates an involvement of another kinase, such as JNK that is described as the main MAP kinase that activates c-Jun [37,38,39]. In line with this, the phosphorylation of JNK was reduced by C3 after 72 until 120 h in p38 ^–/–^ p38 MEFs. In sum, this data confirms a canonical regulation of c-Jun by JNK (Figure 5). Due to the early appearance of the decreased phospho-c-Jun level after 48 h, C3 had to influence additionally another unidentified kinase or phosphatase of c-Jun.

The phosphorylation of the direct upstream MAPK kinase of JNK, MKK4, was only marginally affected by C3. Therefore, this implies that MKK4 was not involved in the reduced phosphorylation of downstream JNK and c-Jun, so that the JNK activity had to be regulated by MKK7 or MAPK phosphatases [42,43,65,66]. Supporting the role of MKK7, prior studies reported that an activation of JNK pathway by use of stimulation with TNFα or IL-1 does not require necessarily MKK4, but MKK7 is sufficient for JNK phosphorylation in various cell types [43,67,68,69].

Upstream of MKK4/7, the decreased activity of TAK1 after 72 and 96 h in C3-treated p38 ^–/–^ p38 MEFs agrees with the arrest of JNK signaling in TGF-β-stimulated cells co-transfected with a kinase-defective TAK1-mutant [46]. Moreover, the simultaneous impairment of p38 and JNK signaling in TNF- or IL-1β-stimulated TAK1-deficient MEFs is in line with the results [70]. Additionally, the inhibition of p38 and JNK cascades mediated through PP2-dependent TAK1 inactivation in COS7 cells endorses the proposed pathways with TAK1 upstream of MKK3/6-p38 and JNK signaling (Figure 5) [71]. In accordance with the C3-induced reduction of TAK1 phosphorylation, Rho is directly involved in TGF-β1-TAK1-signaling via its effector ROCK in myocardial remodeling [72].

## Conclusion

In the present study, we demonstrated that the inhibition of Rho by C3 was essential for the impairment of cell proliferation in p38 ^–/–^ p38 MEFs. We identified the involvement of TAK1, p38- and JNK-pathway as intermediary signal transduction components that mediated downstream a reduced c-Jun phosphorylation resulting in the C3-induced anti-proliferative effect. Moreover, the C3-mediated alterations of the MKK3/6-p38 and JNK-c-Jun signaling not only depended on Rho inactivation, but also on the presence of p38α in MEFs. Thus, the loss of p38α (p38 ^–/–^ EV MEFs) was responsible for the insensitivity towards the C3-induced reduction of phospho-c-Jun via the signal transduction cascade of the MAPKKKs downstream of Rho. As a result of the lacking susceptibility to Rho-dependent alterations of MAPK signaling pathways, the p38 ^–/–^ EV MEFs were resistant to the C3-mediated anti-proliferative effect.

## Additional File

The additional file for this article can be found as follows:

10.5334/1750-2187-12-1.s1

DOI: https://doi.org/10.5334/1750-2187-12-1.s1

## Abbreviations

C3, C3 exoenzyme from *Clostridium botulinum*

C3-E174Q, enzyme-deficient C3-mutant

ERK, extracellular signal-regulated kinase

JNK, c-Jun N-terminal kinase

MAPK, mitogen-activated protein kinase

MAPKK, also MKK, mitogen-activated protein kinase kinase

MAPKKK, mitogen-activated protein kinase kinase kinase

MEFs, mouse embryonic fibroblasts

TAK1, Transforming growth factor-β-activated kinase 1
