# Supplementary material for: Anti-proliferative Effect of C3 Exoenzyme in Fibroblasts is Mediated by c-Jun Phosphorylation
Source: J Mol Signal. 2017 Apr 3;12:1. doi: 10.5334/1750-2187-12-1 (PMC5630077; doi:10.5334/1750-2187-12-1)
Supplement: Supplementary file 1 [file jms-12-148-s1.pdf]

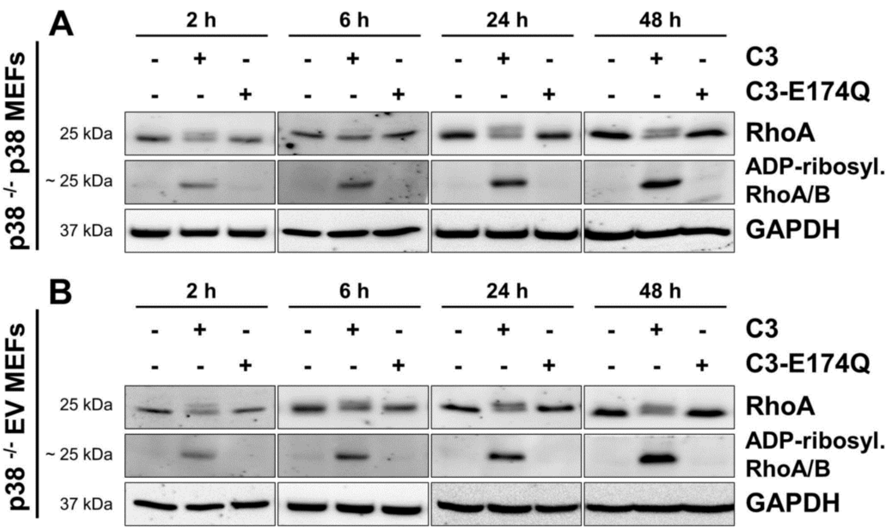

Supplemental Figure S1: C3-mediated ADP-ribosylation of Rho in p38 $^{-/-}$ p38 and p38 $^{-/-}$ EV MEFs. Both cell lines were incubated with 500 nM C3 or 500 nM C3-E174Q for indicated time points. Cells were lysed and applied to Western blot analyses for RhoA, ADP-ribosylated Rho and GAPDH. Representing Western blot analyses of independent experiments (n = 3) for p38 $^{-/-}$ p38 MEFs (A) and p38 $^{-/-}$ EV MEFs (B) are depicted.

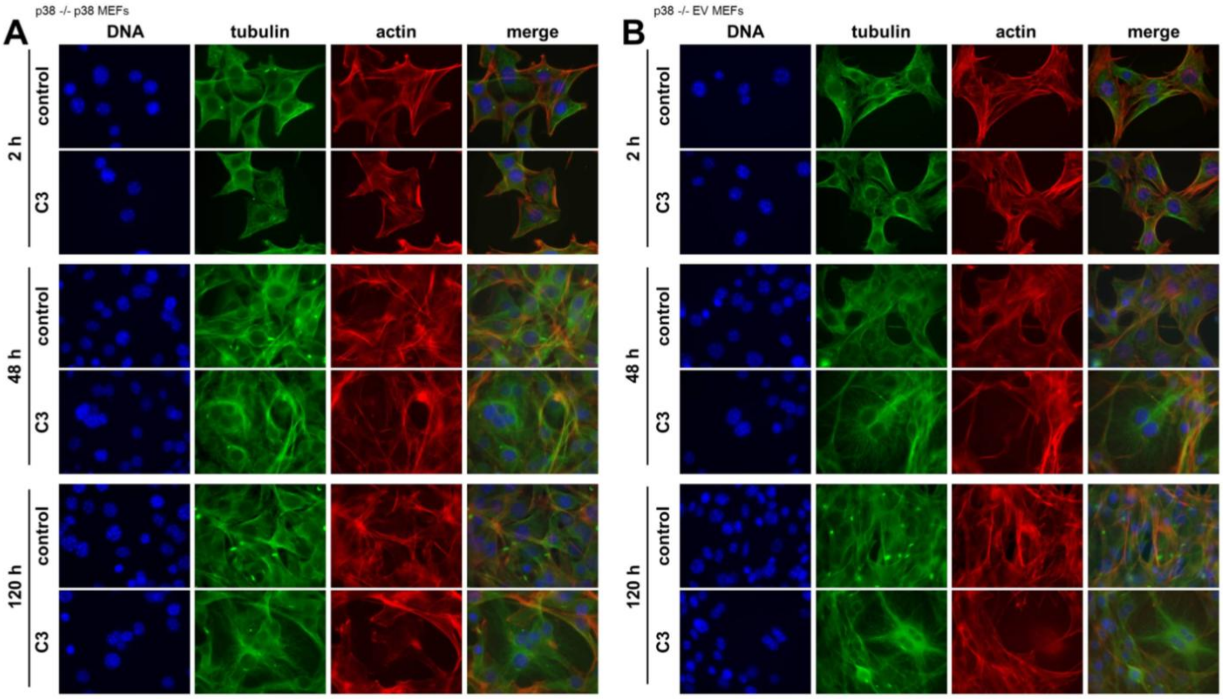

Supplemental Figure S2: C3-mediated morphological changes in p38 $^{-/-}$ p38 and p38 $^{-/-}$ EV MEFs. (A) p38 $^{-/-}$ p38 and p38 $^{-/-}$ EV MEFs (B) were seeded onto coverslips and treated with 500 nM C3 or medium for indicated incubation times. Cells were fixed, permeabilized and unspecific binding sites were blocked. The cell components were stained with DAPI (nuclei), rhodamine/phalloidin (actin) and α-tubulin antibody followed by Alexa Fluor 488-labeled α-rat antibody. Analysis and imaging of morphological alterations was performed by use of fluorescence microscope with a 63x magnification.

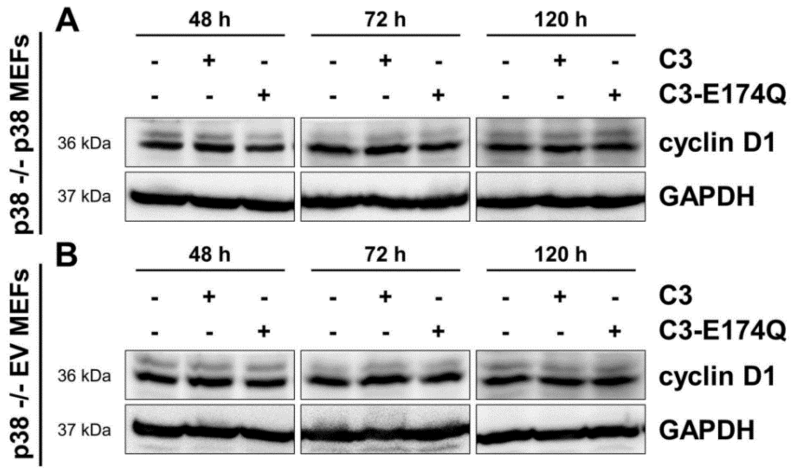

Supplemental Figure S3: Influence of C3 on the abundance of cyclin D1. p38 $^{-/-}$ p38 (A) and p38 $^{-/-}$ EV MEFs (B) were incubated with 500 nM C3 or 500 nM C3-E174Q for indicated time points. Cells were lysed and submitted to Western blot analyses for cyclin D1 and GAPDH. Representing results of independent experiments (n = 3) are shown for both cell lines.

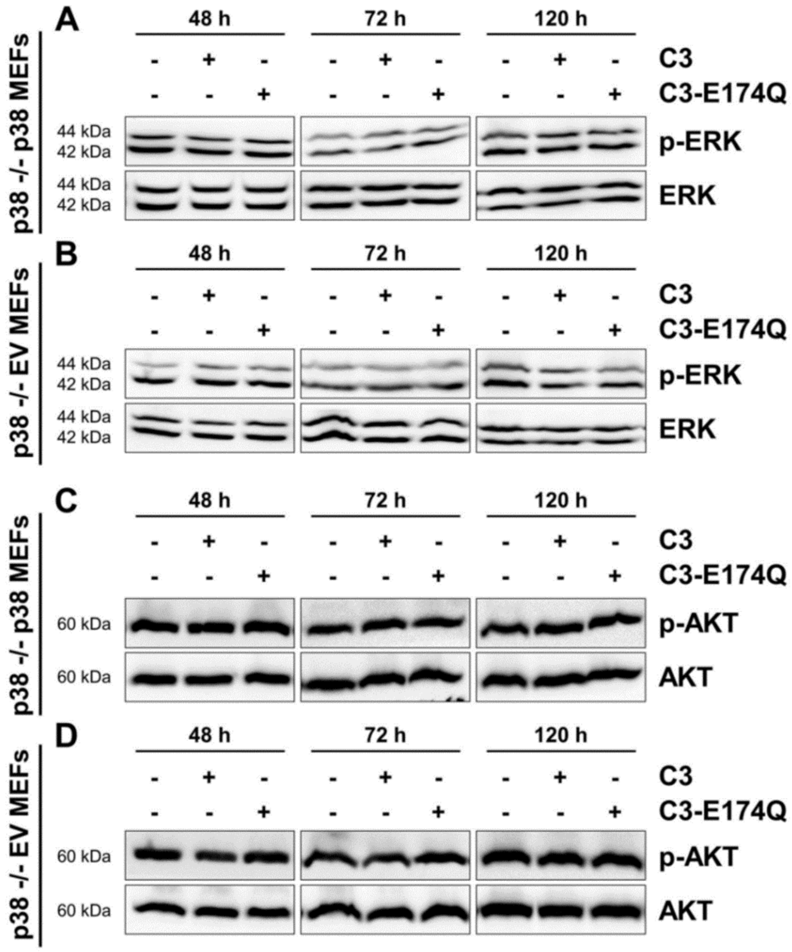

Supplemental Figure S4: Influence of C3 on the phosphorylation of ERK and AKT. After incubation of p38 $^{-/-}$ p38 (A, C) and p38 $^{-/-}$ EV MEFs (B, D) with 500 nM C3 or 500 nM C3-E174Q for indicated time points, cell lysates were submitted to Western blot analyses for phospho-ERK and ERK (A, B), or respectively for phospho-AKT and AKT (C, D). Representing blots of independent experiments (n = 3) are illustrated for both cell lines.

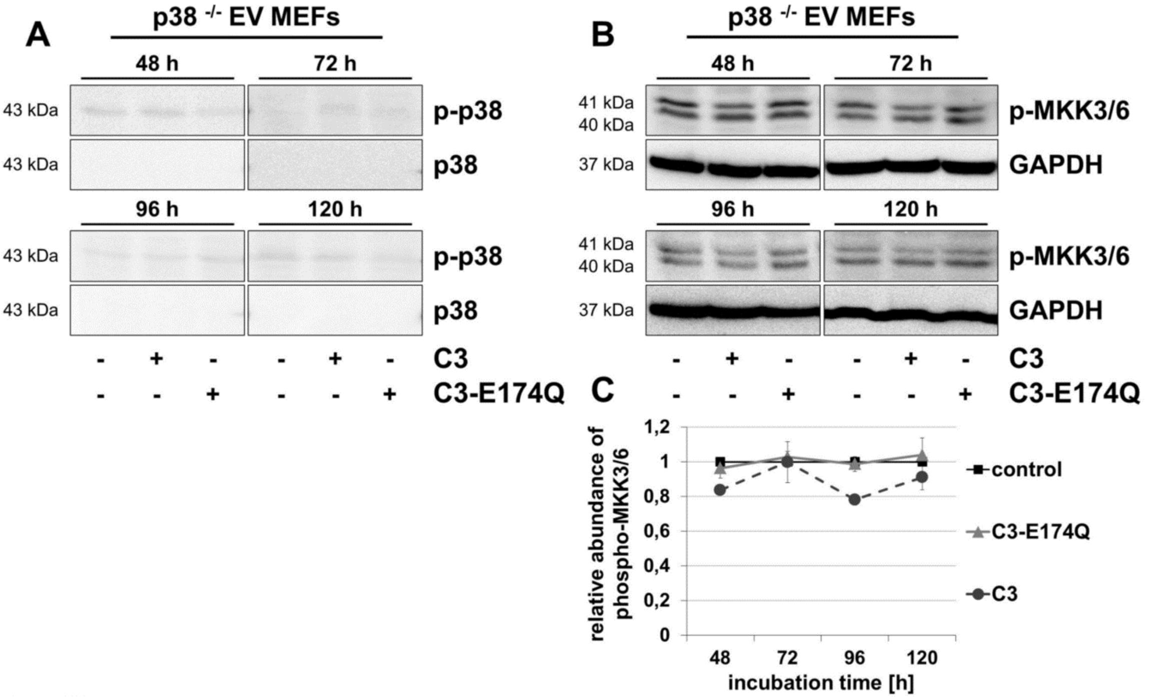

Supplemental Figure S5: Marginal effects on phospho-MKK3/6 in C3-treated p38 $^{-/-}$ EV MEFs. After incubation of p38 $^{-/-}$ EV MEFs with 500 nM C3 or 500 nM C3-E174Q for indicated time points, cells were lysed and submitted to Western blot analyses for phospho-p38 (p-p38) and p38 (A), or phospho-MKK3/6 (p-MKK3/6) and GAPDH (B, C). (C) The densitometric quantification of p-MKK3/6 was performed by normalizing the signal intensity of p-MKK3/6 of C3 and C3-E174Q-treated cells to the signal intensity of control cells. Representative Western blot analyses are depicted. Results represent mean values ± SEM of independent experiments (n = 3).

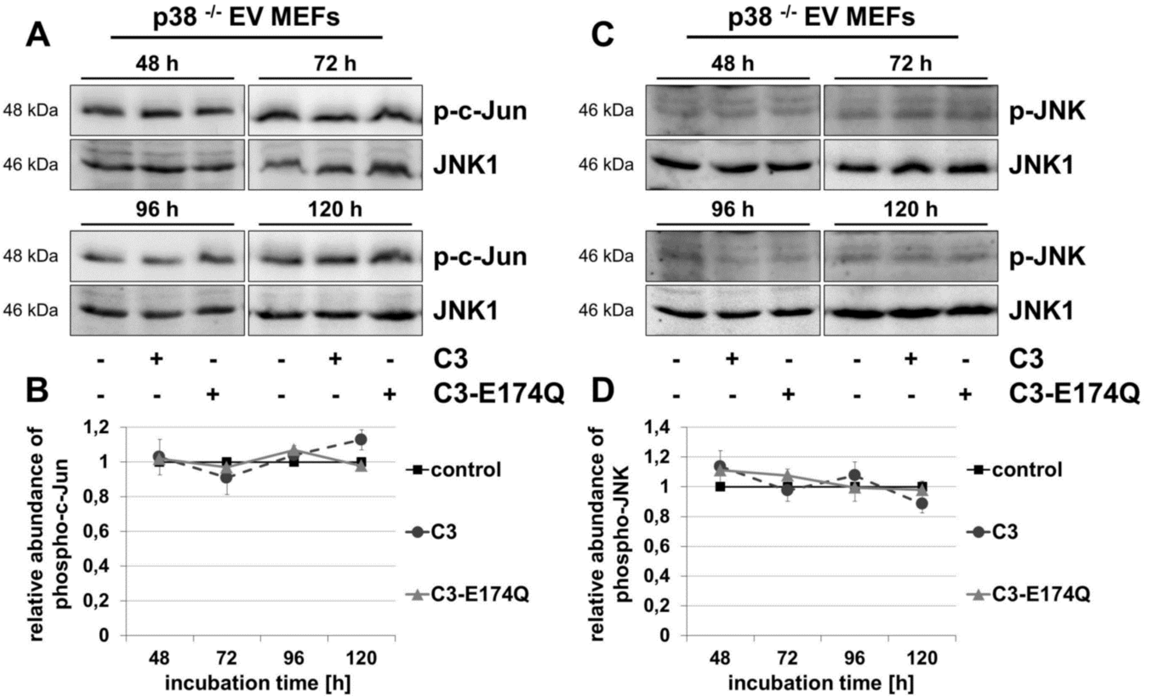

Supplemental Figure S6: C3 did not affect phospho-c-Jun and phospho-JNK in p38 $^{-/-}$ EV MEFs. p38 $^{-/-}$ EV MEFs were treated with 500 nM C3 or 500 nM C3-E174Q for indicated incubation times, lysed and applied to Western blot analyses for phospho-c-Jun (p-c-Jun) (A, B), or phospho-JNK (p-JNK) (C, D) and JNK1. For densitometric analysis the signal intensity of p-c-Jun (B), or respectively p-JNK (D) were normalized to the corresponding intensity of JNK1. Representative Western blot analyses are illustrated. Results represent mean values ± SEM of independent experiments of p-c-Jun (n = 3) and p-JNK (n = 4).

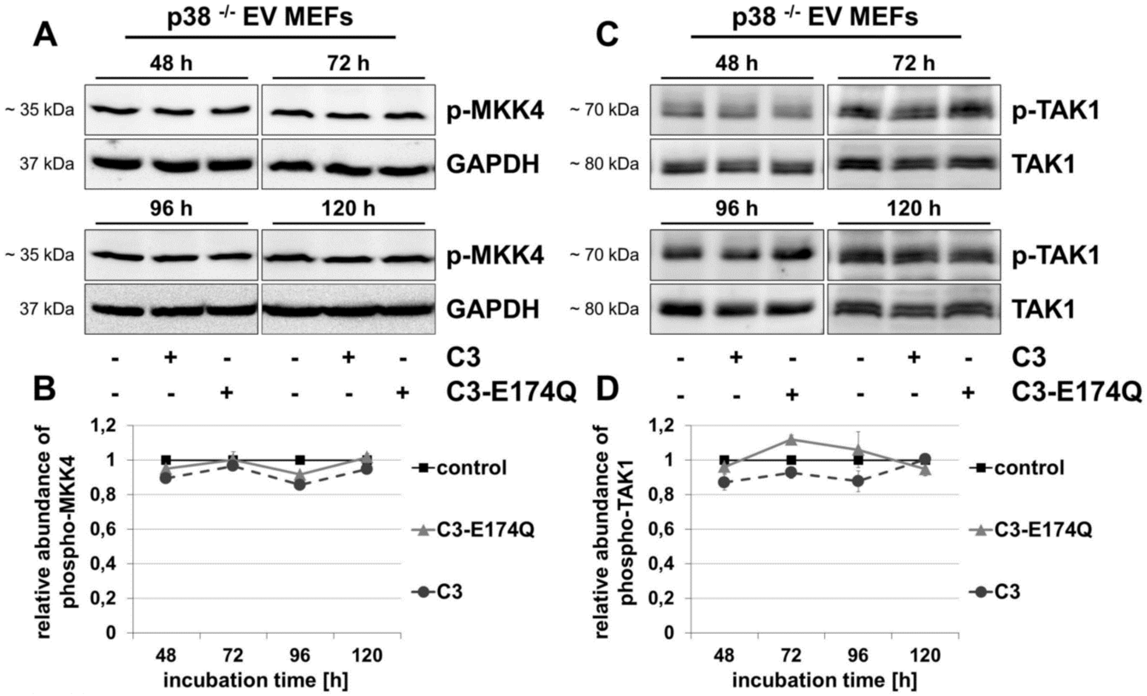

Supplemental Figure S7: Marginal effect of C3 on phospho-MKK4 and phospho-TAK1 in p38 $^{-/-}$ EV MEFs. p38 $^{-/-}$ EV MEFs were treated with 500 nM C3 or 500 nM C3-E174Q for indicated incubation times, lysed and applied to Western blot analyses for phospho-MKK4 (p-MKK4) and GAPDH (A, B), or phospho-TAK1 (p-TAK1) and TAK1 (C, D). (B) For densitometric quantification of p-MKK4, the signal intensity of p-MKK4 of C3- or C3-E174Q-treated cells were adjusted to the corresponding intensity of untreated control cells. (D) For densitometric analysis of p-TAK1 the signal intensity of p-TAK1 were normalized to the signal intensity of TAK1. Representing Western blot analyses are shown. Results illustrated mean values ± SEM of independent experiments of p-MKK4 (n = 4) and p-TAK1 (n = 3).
